# Prevalence of digital amnesia, somatic symptoms and sleep disorders among youth during COVID-19 pandemic

**DOI:** 10.1016/j.heliyon.2022.e10026

**Published:** 2022-07-21

**Authors:** S. James Robert, S. Kadhiravan

**Affiliations:** Department of Psychology, Periyar University, Salem, Tamil Nadu, 636 011, India

**Keywords:** Digital amnesia, Google effect, Somatic symptoms, Sleep disorders, COVID-19, Youth

## Abstract

**Background:**

The proliferation of COVID-19 radically altered people’s daily routines over the last two years, particularly among young. Closures of schools and colleges resulted in virtual learning that increased reliance on gadgets causing digital dependency among youth. The prevalence of digital amnesia, somatic symptoms and sleep disorders among youth during this pandemic require considerable attention since it has not been addressed widely.

**Methods:**

Cross-sectional study was carried out among 326 youth aged between 18 to 25 years. Digital Amnesia Scale, Somatic Symptom Disorder-B Criteria Scale (SSD-12) and Sleep disorders Symptom Checklist (SDS-CL-17) were used to collect data from participants.

**Results:**

Significant positive relationship was found between digital amnesia, somatic symptoms and sleep disorders among youth. Youth differed significantly in their somatic symptoms based on demographic variables such as gender, family type and area of residence. Digital amnesia had significant impact on somatic symptoms through the mediation effect of insomnia and circadian rhythm dimensions of sleep disorders.

**Conclusion:**

Productive use of digital devices would help youth reduce digital amnesia. Practicing digital break/digital detox could also help them improve their cognitive, affective and behavioural aspects, as well as their quality of sleep.

## 1 Introduction

Coronavirus disease 2019 (COVID-19) is an infectious disease caused by the severe acute respiratory syndrome coronavirus 2 (SARS-CoV-2), a respiratory pathogen with high communicability and pathogenicity (World Health Organization, 2020a; World Health Organization, 2020b). COVID-19 is caused by SARS-CoV-2, a novel virus, which belongs to the Coronaviridae family of viruses (Su et al., 2016). The local disease expanded globally, causing a pandemic that affected more than 200 countries. On March 11^th^ of 2020, the World Health Organization (WHO) declared COVID-19 a global pandemic (World Health Organization, 2020a; World Health Organization, 2020b). The COVID-19 epidemic is seriously affecting the nation, society, and people, having an impact on the business sector and the economy as well as people’s jobs, everyday lives, and academic pursuits (Chen et al., 2020; Zhong et al., 2020). India is now the second-highest country affected by the pandemic after the USA with more than 9.4 million confirmed cases and more than 140,000 deaths (Chhetri et al., 2021). Education is one of the many industries that this pandemic has impacted due to this pandemic (UNESCO, 2020). Colleges and universities, among other educational institutions, were closed to students to prevent the spread of COVID-19, thereby obstructing regular educational activities. In India, the fourth phase of lockdown, which was announced or implemented, has had a devastating effect on over a million students. As a result, students from all over the world, including India, began reading and learning at home using digital materials instead of traditional face-to-face teaching method (Jadhav et al., 2020). Although students' engagement with their virtual learning helps them in their academics, it could also result in over-reliance on digital devices, in turn to digital amnesia. Researchers investigated the effect of technology on youth, and coined the term ‘Google Effect’ (Sparrow et al., 2011) whereas "Google effect" and "digital amnesia" can both be interchanged (Lodha, 2019).

### 1.1 Digital amnesia

The term "Digital Amnesia," which was first used by Kaspersky Lab, 2015, refers to the experience of forgetting information that individuals believe a digital gadget will store and recall for them. Digital amnesia and google effect are the results of individuals' dependence on the internet, often systematically considered in the context of an activity approach in experimental psychology (Camerer et al., 2018; Friede, 2013; Molchanov et al., 2018). The over-reliance on the digital devices may negatively affect an individual’s memory that would raise the spectre of digital amnesia. Yale Tribune (2018) claimed that over-reliance on smartphones makes individuals believe that they serve as an alternative to individuals' memories. Apart from adverse effects to the memory, digital amnesia causes disruptive behaviour like a lack of focus, impatience, and increased aggression (Lodha, 2019). Anxiety, depression, posttraumatic stress disorder, and panic attacks have also been associated with digital amnesia (Lodha, 2019). These behavioural and psychological symptoms appeared in people who had either misplaced their smartphones, which contained a flood of their personal and professional information, or whose data had been stolen as a result of malicious third parties (Bhargava, 2016).

Excessive use of technology might lead people to over-reliant on it and impair their capacity to recall things on their own. Even, Socrates was sceptical about technology of his time and warned against “the technology of writing things down” would cause forgetting (Plato, 1925). Similar concerns might be heard in more recent cautions that an over-reliance on technology could result in "digital amnesia" (Gilbert, 2019). Researchers looked into how people choose between using their memories and outside reminders and found that individuals utilize memory tools when they think they might otherwise forget (Gilbert, 2015; Risko and Dunn, 2015). The degree of confidence that each individual has in their memory varies, and these variations produce stable biases regarding whether an individual prefers to utilize their own memory or an external tool (Gilbert et al., 2019).

Smart phones are great and a wonderful technology where everyone should enjoy its benefits in a useful manner; otherwise it can lead to multiple health hazards including digital amnesia (Rani and Roshan, 2020). Smartphones have become indispensable, especially in light of the COVID-19 pandemic quarantine, and without smartphone, one feels incomplete. There has been a growth in smartphone use across a variety of demographics, especially among youth who are attached to their smartphones as a result of COVID-19’s social alienation (Rani and Roshan, 2020).

### 1.2 Google effect

The "Google effect" is the tendency to forget information that can be easily be obtained online using search engines (Kaspersky Labs and Opinion Matters, 2015). Sparrow et al. (2011) addressed the influence of google effect on how people remember, recall and retrieve information. Reliance on search engines for any information is a natural tendency in a society that is becoming more digital and linked. Instead of remembering the actual content, people frequently improve their transactive memory (remembering where to get information online). Every time someone successfully and conveniently retrieves information from external storage, the transactive memory is strengthened, and it is now more convenient to use external information storage instead of making room in one’s memory for such information. As such, digital devices and the internet serve as an external memory storage, an alternative for memorizing information (Hassani et al., 2021). A study by the Kaspersky Lab, 2017 discovered that roughly 30% of people forget an online fact shortly after accessing it. Digital gadgets and the internet are used for remembering and disclosing information that can be easily obtained online.

### 1.3 Digital amnesia and somatic symptoms

There has been a significant surge in usage of digital devices due to online learning platforms since COVID-19 pandemic (Strielkowski, 2020). Youth started using digital devices extensively to resume their studies through online, consequently resulting in digital dependency. Our ability to think, recall, pay attention, and control our emotions is being negatively impacted by our dependence on smartphones and other digital devices rather than being aided (Morin, 2013). Digital dependency may have detrimental repercussions on a person’s development, including physical inactivity (Griffiths et al., 2010), emotional instability (Manganello and Taylor, 2009), sleep disorders, and impaired memory (Dworak et al., 2007). Gunes (2020) reported that people played video games for two hours a day on the weekends, which is not only bad for their physical health (Rosen et al., 2014) but also has effects on their social and emotional development (Hu et al., 2020a, 2020b; Mistry et al., 2007), cognitive growth (Kumari and Ahuja, 2010), and attention span (Swing et al., 2010). Digital dependency to an extent causes cognitive impairments (Seki et al., 2019), emotional instability (Xiuqin et al., 2010) and sleep disorders (Salicetia, 2015).

### 1.4 Digital amnesia and sleep disorders

Digitalization is widespread and the most accessible approach to staying in touch with loved ones despite the social isolation during the COVID-19 pandemic. However, limitless utilization of these digital gadgets may further develop sleep disorders. Over-dependence on digital devices during the pandemic might cause sleep deprivation and multiple psychological problems. As a result of disruptions to sleep and decreased synaptic pruning, digital amnesia impairs the ability to develop and maintain new memories and hinders the ability to recall new information. It is reported that excessive use of smartphones during the day, increase the likelihood of sleep disorder (Thomee et al., 2011). People are utilizing digital devices more than ever before which is causing sleep deprivation in the form of later bedtimes, shorter sleep durations, and longer sleep onset latency. The addiction to digital devices may affect the quality of sleep due to later bedtimes and shorter sleep durations. These adverse associations between excessive use of digital devices and sleep quality also include time displacement, sleep physiology, psychological stimulation based on media content, and the impact of light emitted from devices on circadian timing (Arora et al., 2021).

The symbiotic living with digital devices has changed the way individuals think, learn, remember, and behave (Kaspersky Labs and Opinion Matters, 2015) especially during this COVID-19 pandemic. The COVID-19 lockdown has given a valid reason for people, particularly youth, to be locked to their digital devices such as smart phones. Smartphone is one thing that individuals never let beyond their sight; it is always available either in their hand or within a touchable distant. Youths are regularly active on various social media platforms to socialize with peers and others which may lead to digital dependency. With online learning becoming the norm as part of the digital transformations, they have all the more reasons to be glued to their phones that would obviously lead to digital dependency causing digital amnesia.

The COVID-19-specific risk factors for sleep disorders include anxiety about the disease, uncertainty about treatment and prevention strategies, and a negative attitude toward control measures (Tasnim et al., 2020). The number of global deaths due to COVID-19 predicted more somatic complaints and worse sleep quality (Simor et al., 2021). People’s mental health has significantly suffered as a result of the COVID-19 pandemic and associated quarantine procedures (Torales et al., 2020; Rossi et al., 2020). Furthermore, anxiety, depression, insomnia, somatic disorder, and irritability have been related to quarantine (Hossain et al., 2020; DiGiovanni et al., 2004; Hawryluck et al., 2004). Hence, it is the need of hour to investigate the prevalence of digital amnesia, sleep disorders and somatic symptoms among youth.

## 2 Methods

### 2.1 Participants and study design

A descriptive survey was utilized to study the prevalence of digital amnesia, somatic symptoms, and sleep disorders among young population during the pandemic. The population of the study comprised of 327 youth aged 18 to 25, selected through incidental sampling. All the three scales were tested for normality. It was found that the Skewness and Kurtosis values are ranged between +2 and − 2 which showed that the data was normally distributed, hence parametric tests were utilized for statistical analysis.

### 2.2 Procedure

The researchers approached participants through WhatsApp after receiving permission from the college authorities. Participants were then given information about the study with informed consent, and interested people only were provided with questionnaires in the Google form. They were also given with the option to discontinue the study at any point of time, as well as assurance of confidentiality.

### 2.3 Digital amnesia scale

The researchers developed the digital amnesia scale to assess the level of digital amnesia. The items were derived from the case study conducted by Musa and Ishak (2020). In the current study, the items (eg. “I trust my smartphones to store data that I cannot do with my own memory”) were rated on a 5-point Likert scale ranging from 1 to 5. The overall score ranged between 1 and 50. In this study, the reliability of the scale was established using Cronbach alpha 0.71, indicating an acceptable level of internal consistency (Tavakol and Dennick, 2011) with satisfactory validity.

### 2.4 Somatic Symptom Disorder-B Criteria Scale (SSD-12)

SSD-12 was developed by Toussaint et al. (2016). It is used to assess cognitive, affective and behavioral aspects of somatic symptoms. It has 12 items with a score of 0–4. The overall score ranges from 0 to 48. Construct validity was examined using bivariate correlations between SSD-12, PHQ-15, GAD-7, and WI-7 by the authors (Toussaint et al., 2016) and found to have satisfactory validity. The Cronbach alpha for cognitive, affective and behavioural dimensions were 0.94, 0.71 and 0.92 respectively (Toussaint et al., 2016).

### 2.5 Sleep disorders Symptom Checklist-17

It was developed by Klingman et al. (2017). It is used to assess dimensions of sleep disorders such as insomnia, obstructive sleep apnea, restless legs syndrome, circadian rhythm, narcolepsy, and parasomnias. It has 17 items with a score of 0 (never) to 3 (frequently). The total score ranges from 0 to 51. The content validity was established by the authors and the Cronbach alpha for insomnia, obstructive sleep apnea, circadian rhythm, restless legs syndrome, narcolepsy and parasomnias were 0.76, 0.69, 0.93, 0.80, 0.93 and 0.83 respectively (Klingman et al., 2017).

### 2.6 Ethics

The Declaration of Helsinki was followed when conducting the study and approved by the local ethical committee. All participants provided their written informed consent.

## 3 Results

A total of 327 youth participated in the online study. Among them 111 (34%) were boys and 216 (66%) were girls aged between 18 and 25 years (M = 22.12, SD = 5.36). Correlation matrix for study variables were given in Table 1. The analysis showed that digital amnesia was positively correlated with somatic symptoms (0.304) and dimensions of sleep disorders such as insomnia (0.280), circadian rhythm (0.257), narcolepsy (0.230), obstructive sleep apnea (0.301), restless legs syndrome (0.238) and parasomnias (0.331).

**Table 1:** Correlation matrix for the study variables.

Variables	M	S.D	1	2	3	4	5	6	7
1. Digital amnesia	33.85	5.734	–						
2. SSD	13.27	10.381	.304∗∗	–					
3. SD-Insomnia	3.39	2.820	.280∗∗	.559∗∗	–				
4. SD-Circadian rhythm	1.79	1.354	.257∗∗	.254∗∗	.287∗∗	–			
5. SD-Narcolepsy	1.16	1.299	.230∗∗	.554∗∗	.543∗∗	.343∗∗	–		
6. SD-Obstructive sleep apnea	1.98	2.243	.301∗∗	.655∗∗	.660∗∗	.342∗∗	.638∗∗	–	
7. SD-Restless legs syndrome	1.59	2.107	.238∗∗	.538∗∗	.539∗∗	.308∗∗	.630∗∗	.653∗∗	–
8. SD- Parasomnias	1.72	1.931	.331∗∗	.590∗∗	.529∗∗	.301∗∗	.572∗∗	.665∗∗	.676∗∗

P < 0.01; SSD- Somatic Symptom Disorder; SD-Sleep disorders.

Table 2 shows the significant difference in somatic symptoms among youth on the basis of their gender, family type and area of residence. Somatic symptoms were significantly higher among boys than girls (16.78 vs 11.47). Students from joint families were at more risk of developing somatic symptoms than students from nuclear families (16.31 vs 11.95). Furthermore, students from urban area had a higher level of somatic symptoms compared to students from rural area (14.89 vs 11.90).

**Table 2:** Differences in somatic symptoms by demographic categories.

Variables	Category	N	Percentage (%)	M	SD	t-value
Gender	Boys	111	34.0	16.78	11.104	4.514∗
	Girls	216	66.0	11.47	9.522	
Family type	Nuclear	228	69.6	11.95	9.453	3.552∗
	Joint	99	30.4	16.31	11.750	
Area of residence	Rural	177	54.0	11.90	8.954	2.612∗
	Urban	150	46.0	14.89	11.671	

P < 0.05.

**Figure 1:**
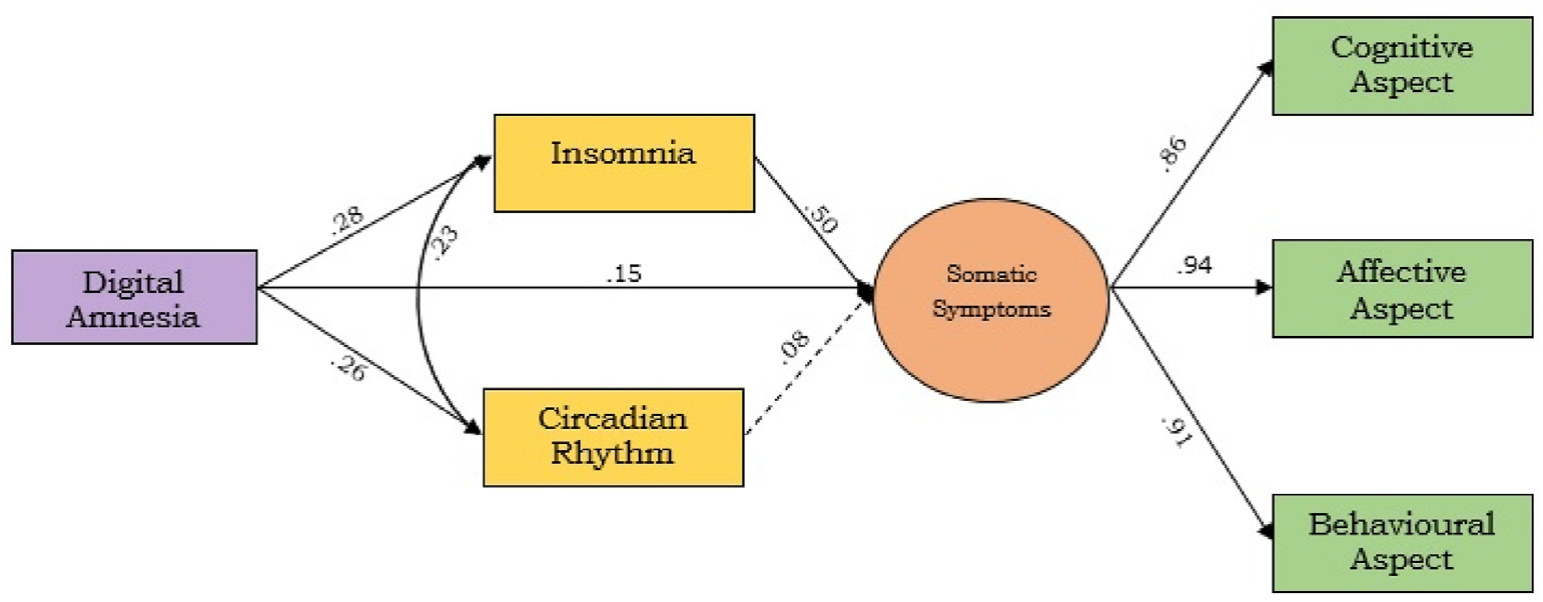
Structural equation modeling diagram for standardized path. Note: Solid lines represent significant path and dotted line represents non-significant path.

Structural equation modeling was carried out to analyse the model fit for somatic symptoms as outcome variable, incorporating the selected predictor factors. In the model, digital amnesia was considered as exogenous variable. Insomnia and circadian rhythm of sleep disorders were considered as mediators of digital amnesia and somatic symptoms. All the variables were analysed for satisfactory fit, and it was found that all of them were within the acceptable values: CFI = 0.997; GFI = 0.991; AGFI = 0.969; RMSEA = 0.040 and SRMR = 0.012. We pruned the model by deleting non-significant and weak factors to increase the model’s significance and overall fit (Hooper et al., 2008). As such, we removed four factors from sleep disorders (Narcolepsy, Obstructive sleep apnea, Restless legs syndrome and Parasomnias). Table 3 represents the model fit and path analysis diagram is shown in Figure 1.

**Table 3:** Model fit summary and structural models comparison (N = 326).

Indices	Obtained Values	Suggested values	References
Chi-Square	1.524	<5	Kline, 2015
GFI	0.991	>0.90	Hair et al., 2006
AGFI	0.969	>0.90	Daire et al., 2008
CFI	0.997	>0.90	Hu and Bentler, 1999
SRMR	0.012	<0.08	Hu and Bentler, 1999
RMSEA	0.040	<0.08	Hair et al., 2006

This model provided additional insight into the findings of correlation which suggested a causal and structural relationship among the variables. It is apparent from the model that the role of insomnia was stronger in predicting somatic symptoms (0.50) compared to digital amnesia (0.15) and circadian rhythm (0.08). Digital amnesia also showed a positive significant effect on insomnia (0.28) and circadian rhythm (0.26). Finally, the overall effect of digital amnesia on somatic symptoms through the mediation of insomnia and circadian rhythm was significant and positive (see Table 4).

**Table 4:** Effect of exogenous factors on somatic symptoms: direct, indirect and overall effect.

Effect	Digital amnesia	Insomnia	Circadian Rhythm
Direct	0.153	0.505	0.081
Indirect	0.162	-	-
Total	0.315	0.505	0.081

## 4 Discussion

The present study shed light on the prevalence of digital amnesia, somatic symptoms and sleep disorders among youth during COVID-19 pandemic. Youth having a higher level of digital amnesia were likely to develop more somatic symptoms as well as insomnia, obstructive sleep apnea, circadian rhythm, restless legs syndrome, narcolepsy, and parasomnias of sleep disorders. Insomnia is a sleep disorder that causes difficulty falling and/or staying asleep. The condition can be short-term (acute) or long-term (chronic). Narcolepsy refers to the condition characterized by excessive daytime sleepiness interspersed with brief "attacks" of sleep during waking hours. This kind of sleep attacks can happen at any moment or during any activity, including potentially risky situations such as driving a car. Obstructive sleep apnea is the sudden stopping of breathing during sleeping that happens as a result of an obstruction in the upper airway or a failure of the respiratory centres in your brain to activate breathing. Parasomnias is a sleep disorder characterized by abnormal behaviour or physiological events while sleeping or in the transitional state between sleep and waking. Poor or inadequate sleep would have affected the individuals' cognitive, affective and behavioural aspects leading to somatic dysfunction.

The pandemic and ensuing lockdown resulted in a greater reliance on digital gadgets, which had a negative impact on health by disrupting sleep patterns and duration, as well as leading to the development of somatic symptoms (Majumdar et al., 2020). Lam (2014) reported that over-reliance on gadgets can significantly impact the sleep-wake cycle, resulting in insomnia and other sleep difficulties which is consistent with the current findings. It was also noticed that youth with sleep problems have more somatic symptoms. People with insomnia tend to report a higher risk of developing somatic symptoms (Wong et al., 2015). Huang et al. highlighted that sleep problems such as insomnia was closely related to somatic symptoms (Huang et al., 2020) which is consistent with present finding.

A highly significant difference in somatic symptoms was found between boys and girls, which was higher among boys. Previous study has shown that somatic symptoms were more common in boys who were excessively reliant on gadgets (Cao et al., 2011) which supports the present findings. A contradictory result was found by Cerutti et al. (2016) who reported that girls consistently scored higher than boys in somatic symptoms (M = 16.53, SD = 11.73 vs M = 11.98, SD = 10.34).

The structural equation modeling shed light on the effect of digital amnesia on somatic symptoms through the mediating role of insomnia and circadian rhythm of sleep disorders. Digital amnesia had a direct effect on both dimensions of sleep disorders (insomnia and circadian rhythm) as well as on somatic symptoms. Digital amnesia and insomnia demonstrated a positive and significant effect on somatic symptoms. Poor quality of sleep, difficulty in falling asleep or staying asleep mediated the effect of digital amnesia on somatic symptoms were reported by youth participated in the present study which indicates that there was a strong indirect influence of digital amnesia on somatic symptoms through the mediation of insomnia and circadian rhythm than the direct influence. Specifically, the findings of the current study demonstrated that digital amnesia increased the risk of poor quality of sleep, which could increase the susceptibility to somatic symptoms. This type of mediation of sleep issues had been mentioned in earlier studies. For example, Cerutti et al. (2021) found that sleep disturbances partially mediated the association between over-reliance on digital devices and somatic symptoms. The quality of sleep has an impact on how people perceive the world, and getting disrupted sleep makes them pessimistic (Jiang et al., 2017) which may increase their risk of developing somatic symptoms.

## 5 Conclusion

The utilization of digital devices are unavoidable in the modern world and it is completely based on the self-comfort of individuals. The usage of digital gadgets productively and efficiently has become the need of the hour, especially during this pandemic. Proper awareness program has to be organized for youth on the productive use of digital devices which would help them to overcome digital amnesia. Boys could be sensitized about the negative impact of digital amnesia, and utilize the digital devices only when it is necessary.

Youth should be encouraged to practice digital break everyday by disconnecting from the online world and focus on other healthy lifestyle such as doing physical activities, and adequate time with family. These health-promoting behaviours might help them diminish digital amnesia and improve their sleep quality. They can also achieve harmony between their online and offline activities by taking a brief break from their online platform. The better students understand their strengths and limitations of usage of digital devices, the better they will be able to master their gadgets. Moreover, having a practice of sleeping consistently would improve their cognitive, affective and behavioural aspects.

## 6 Benefits

The results of this study could be used to sensitize youth about the detrimental effects of digital amnesia on sleep quality as well as cognitive, emotional, and behavioural components. Youth could benefit from regular training programmes that educate them about digital amnesia and how to handle it efficiently. Additionally, youth could be educated to practise digital break for the improvement of their physical and psychological wellbeing.

## 7 Recommendations for practice

The digital amnesia scale could be expanded with different dimensions in the future study for different target populations. The role of digital amnesia can be further studied examining other variables such as digital intelligence, personality traits and emotional intelligence. The concept of digital amnesia could be explained in learning perspective such as reinforcement theories. Information processing approach of memory may also be incorporated with digital amnesia in future studies.

## Declarations

### Author contribution statement

S. James Robert: Conceived and designed the experiments; Performed the experiments; Analyzed and interpreted the data; Contributed reagents, materials, analysis tools or data; Wrote the paper.

S. Kadhiravan: Conceived and designed the experiments; Analyzed and interpreted the data; Contributed reagents, materials, analysis tools or data; Wrote the paper.

### Funding statement

This research did not receive any specific grant from funding agencies in the public, commercial, or not-for-profit sectors.

### Data availability statement

Data associated with this study has been deposited at https://docs.google.com/spreadsheets/d/11AIAkjpF2DUA31fO%209i8YFp0amKQ09YA/edit?usp=sharing&ouid=104246682839958167367&rtpof=true&sd=true

### Declaration of interest’s statement

The authors declare no conflict of interest.

### Additional information

No additional information is available for this paper.
